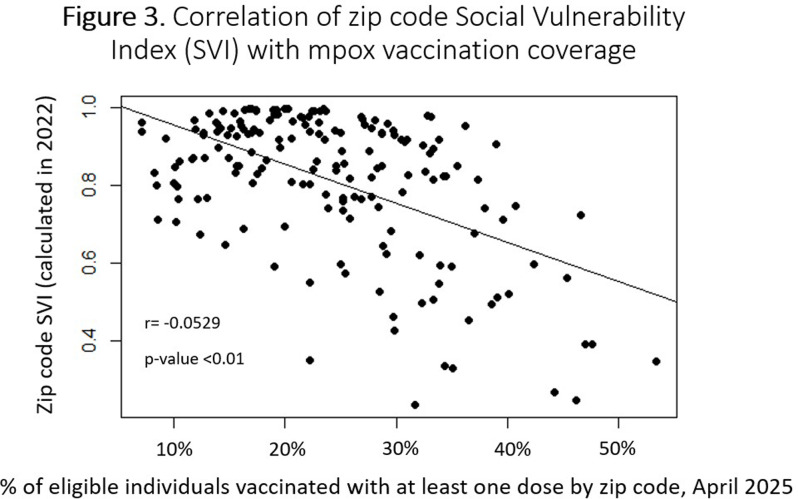# 59 Clear After a Year? Insights from a Pediatric Approach to Contact Precaution Discontinuation for Resistant Gram-Negatives Organisms

**DOI:** 10.1017/ash.2026.10490

**Published:** 2026-06-23

**Authors:** Anthony Lo Piccolo, Emma Kaplan-Lewis, Kruti Gala, Eunice Casey, Gabriel Cohen, Stephanie Rasmussen, Andrew Wallach, Ofole Mgbako, Justin Chan

**Affiliations:** 1 NYU Langone Health; 2 NYC Health and Hospitals; 3 NYC Health + Hospitals; 4 NYC Health + Hospitals/Bellevue; 5 NYU Grossman School of Medicine

## Abstract

**Background:** NYC Health + Hospitals (H+H) provides care to large communities experiencing social vulnerability. During the 2022 mpox outbreak, H+H implemented a large-scale, targeted mpox vaccination campaign using electronic health record (EHR) reminders among groups with greatest risk of mpox infection. When demand was high, 25% of available vaccine appointments were reserved for established H+H patients. This study aims to describe patterns of mpox vaccination amongst eligible H+H patient groups. **Methods:** H+H started vaccinating with JYNNEOS in May 2022 and used EHR reminders to encourage vaccination amongst those eligible, including: (1) Patients with an HIV diagnosis AND who had a sexually transmitted infection in the past year; OR (2) Patients who received at least one mpox vaccine and was due for the second; OR (3) Patients with a sexual orientation in the EHR as Gay, Bisexual, Lesbian, Something else, or Choose not to disclose; OR (4) Patients with a gender identity in the EHR as Transgender Female, Transgender Male, Gender Non-binary/Nonconforming, Other, or Choose not to disclose. We extracted data from the NYC Citywide Immunization Registry through April 30, 2025, into our EHR. We calculated a Pearson correlation coefficient between the 2022 Centers for Disease Control and Prevention (CDC) Social Vulnerability Index (SVI) and percentage who received at least one mpox vaccination by zip code with significance set at a p-value <0.05. **Results:** Figure 1 summarizes key events along the H+H mpox vaccination timeline. There were 112,680 H+H patients eligible for mpox vaccination, of which 25.7% received at least one dose by April 2025, compared to 51.5% amongst patients with HIV (Table). White patients had the highest proportion vaccinated (31.1%), and Hispanic/Latinx patients had the lowest (22.0%). Figure 2 indicates the percentage vaccinated by neighborhood at three time points (September 2022, April 2023, April 2025). As of April 2025, the percentage of eligible individuals vaccinated by zip code was negatively correlated with SVI of the zip code (r= -0.0529; p<0.01) (Figure 3). **Conclusion:** Amongst eligible H+H patients, we identified disparities in mpox vaccination rates by race/ethnicity, neighborhood, and the zip code SVI. The use of EHR reminders may have helped us achieve a higher vaccination rate amongst eligible patients with HIV, compared to the overall cohort. A limitation may be misclassification of eligible individuals, which relies on structured social history and gender identity data accurately documented in the EHR. Our findings identify areas for quality improvement to optimize vaccine equity.

**Figure:**
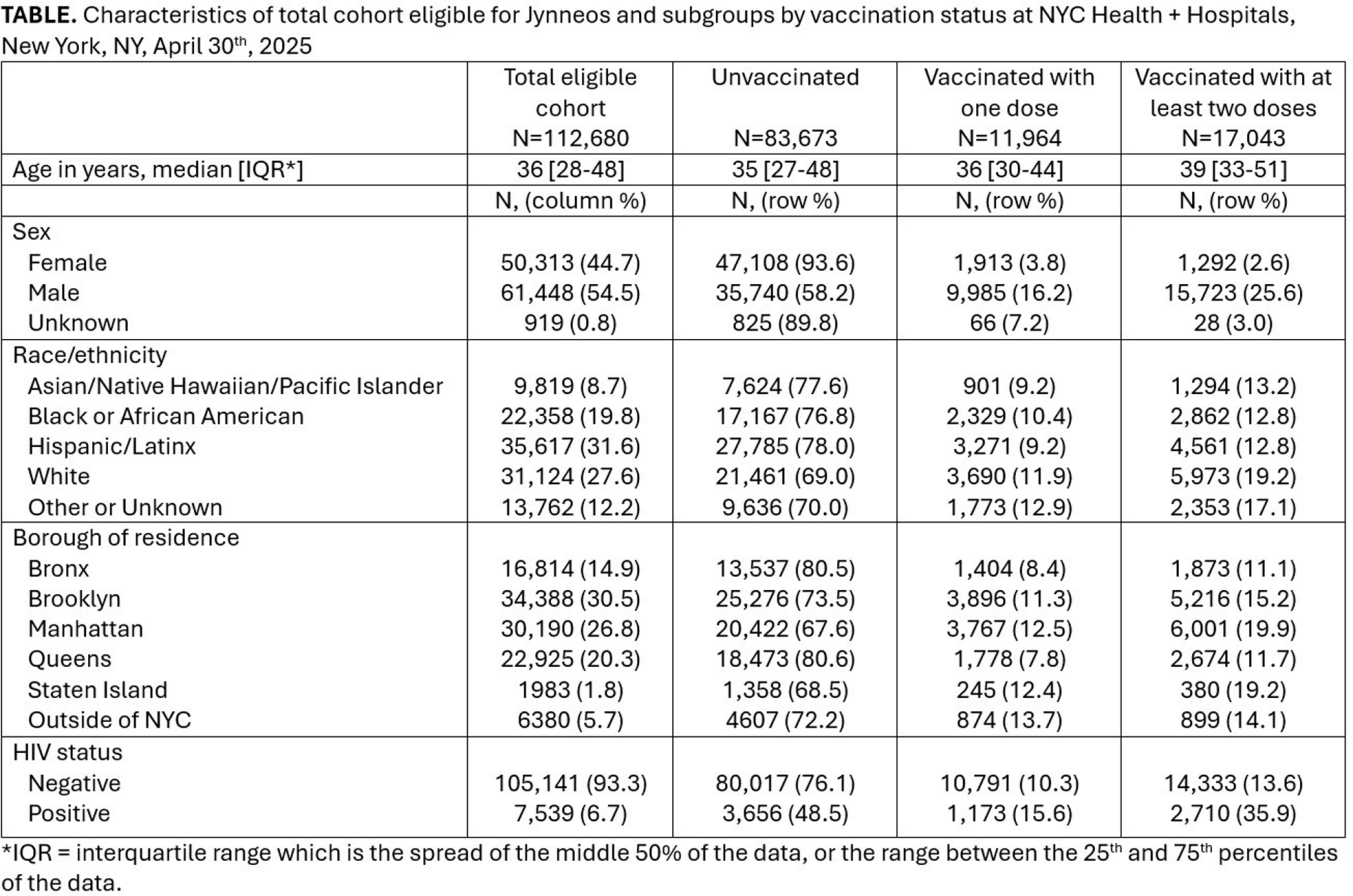

**Figure:**
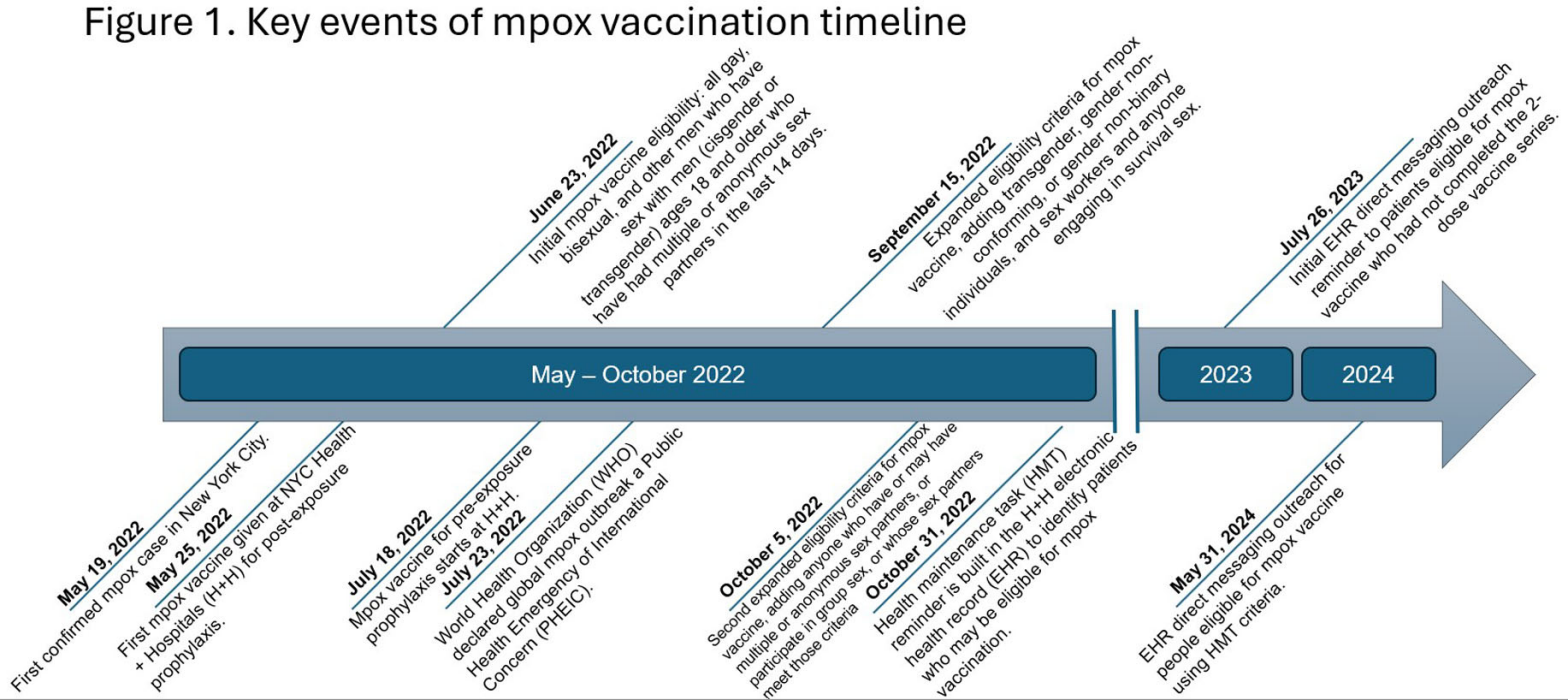

**Figure:**
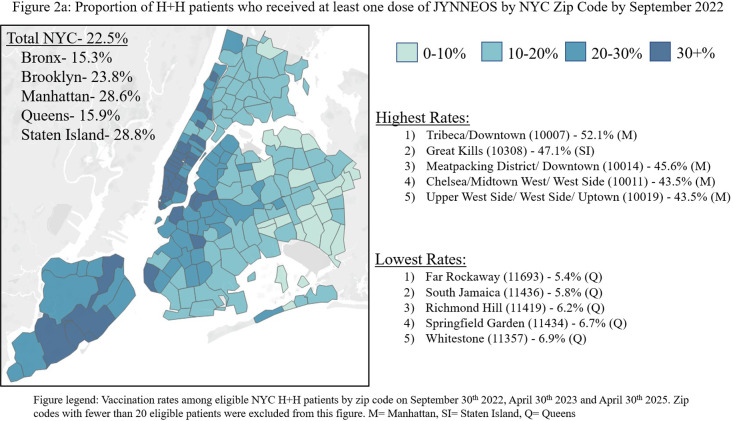

**Figure:**
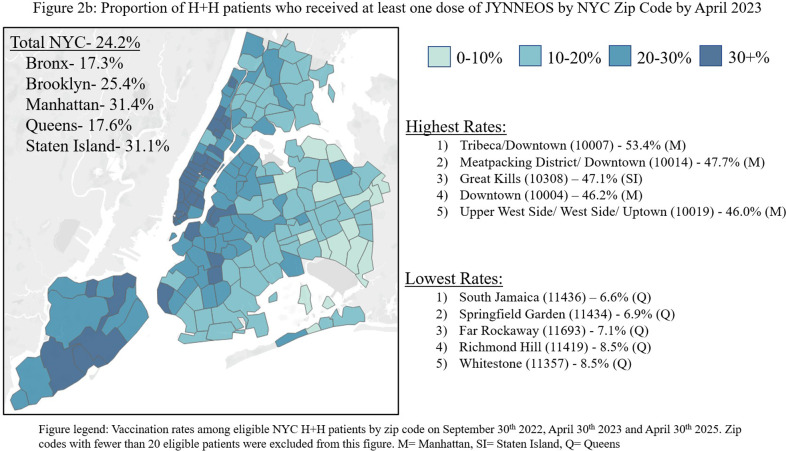

**Figure:**
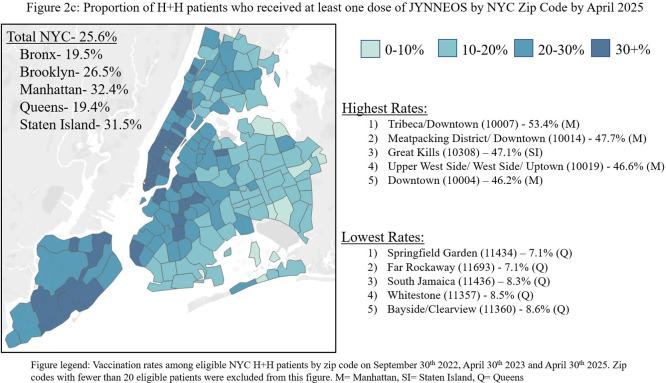

**Figure:**